# c-MET receptor as potential biomarker and target molecule for malignant testicular germ cell tumors

**DOI:** 10.18632/oncotarget.25867

**Published:** 2018-08-07

**Authors:** Katia Corano Scheri, Erica Leonetti, Luigi Laino, Vincenzo Gigantino, Luisa Gesualdi, Paola Grammatico, Mariano Bizzari, Renato Franco, J. Wolter Oosterhuis, Hans Stoop, Leendert H.J. Looijenga, Giulia Ricci, Angela Catizone

**Affiliations:** ^1^ Department of Anatomy, Histology, Forensic-Medicine and Orthopaedics, “Sapienza” University of Rome, Italy; ^2^ Department of Molecular Medicine, Laboratory of Medical Genetics, “Sapienza” University of Rome, San Camillo-Forlanini Hospital, Rome, Italy; ^3^ Pathology Unit, Istituto Nazionale Tumori I.R.C.C.S. "Fondazione Pascale", Naples, Italy; ^4^ Department of Experimental Medicine, Systems Biology Group Lab, “Sapienza” University of Rome, Italy; ^5^ Pathological Anatomy Unit, Department of Psychic and Physic health and preventive medicine, Università degli Studi della Campania “Luigi Vanvitelli”, Naples, Italy; ^6^ Department of Pathology, Laboratory for Experimental Patho-Oncology, Erasmus MC University Medical Center, Cancer Institute, Rotterdam, The Netherlands; ^7^ Department of Experimental Medicine, Università degli Studi della Campania “Luigi Vanvitelli”, Naples, Italy

**Keywords:** TGCTs, c-MET, HGF, c-MET inhibitors, cancer therapy

## Abstract

Type II testicular germ cell tumors (TGCTs) represent the most frequent malignancy in Caucasian males (20–40 years). Even if diagnosed with disseminated disease, >80% of patients are cured; however, a small percentage of cases progress and result in death. It is commonly accepted that these cancers arise from a disturbed testicular embryonic niche that leads to the block of gonocyte differentiation. The subsequent development of the invasive seminomas and non-seminomas is due to a combination of genetic, epigenetic and microenvironment-based alterations (genvironment). Hepatocyte growth factor (HGF) is present in the testicular microenvironment, together with its receptor c-MET, from early embryonic development to an adult stage. In addition, c-MET is a well-known proto-oncogene involved in the onset and progression of various human cancers. Herein, we have investigated the expression and availability of HGF and c-MET in TCam-2, NCCIT and NT2D1 cells, which are type II (T)GCT representative cell lines, and the effect of c-MET activation/repression on the regulation of cancerous biological processes. We found that NT2D1 cells increase their proliferation, polarized migration, and invasion in response to HGF administration. NCCIT cells respond to HGF stimulation only partially, whereas TCam-2 cells do not respond to HGF, at least according to the investigated parameters. Interestingly, the immunohistochemical study of c-MET distribution in TGCTs confirm its presence in both seminoma and non-seminoma lesions with different patterns. Notably, we found the highest c-MET immunoreactivity in the epithelial elements of the various components of TGCTs: teratoma, yolk sac tumor and choriocarcinoma.

## INTRODUCTION

Testicular germ cell tumors are a heterogeneous group of neoplasms with different histopathology and variable clinical course. Histologically and clinically, they are subdivided into type I (infants), type II (adolescents and young adults, i.e., TGCTs) and type III (elderly) [1, 2]. The incidence of type II, by definition malignant tumors (seminomas and non-seminomas), has drastically increased in the last decades [3]. They originate from a common precursor lesion, the germ cell neoplasia *in situ* (GCNIS), which arises from transformed primordial germ cells/gonocytes. The default development of this lesion leads to the formation of seminomas, whose cells present gonocyte-like features. A genetic reprogramming of these cells gives rise to embryonal carcinoma cells, the stem cells of non-seminomas, malignant tumors that mimic embryonic development, both with possible embryonic (teratomas) and extra-embryonic differentiation (yolk sac tumors and choriocarcinomas) [2, 4, 5]. These cancers are mainly characterized by a good prognosis, since they are extraordinarily chemo- and radio-sensitive. However, in a small percentage of cases, a cisplatin-resistance exists, making cure difficult. For this reason, TGCTs remain an important cause of mortality in young men. A deeper investigation of TGCT biology may allow an identification of novel biological therapies or novel predictive markers of an aggressive disease [6–8].

TGCTs are featured by low rates of somatic mutations, which is exceptional for solid cancers in adults [9–15]. Notwithstanding, these cancers present genetic alterations, such as a high frequency of chromatin rearrangement and chromosomal anomalies (among them, chromosome 12 alterations have been fully described) [16–20]. In addition, a gain of chromosome 7, whose region 7q31 encodes the tyrosine kinase receptor c-MET, has been described in TGCTs [21]. However, no c-MET mutations have been reported so far in these cancers [22]. An alteration of the c-MET pathway has been reported in several cancer types [23–25] (www.vai.org/met). It has also been shown that treatment with c-MET selective inhibitors, in both *in vitro* and *in vivo* models, promotes a slow-down of tumor growth [26–28]. As a result, patients are currently recruited for Phase I, II and III anti-tumor clinical trials of these drugs (http://www.clinicaltrials.gov). The c-MET receptor binds to hepatocyte growth factor (HGF), a pleiotropic cytokine produced by mesenchymal cells, which acts on epithelial cells in a paracrine fashion [29–32]. The HGF/c-MET interaction triggers c-MET receptor dimerization and tyrosine phosphorylation, thus modulating multiple biological processes, including proliferation, migration and invasion, morphogenesis and tubulogenesis, differentiation and apoptosis escape [33, 34]. Notably, all these phenomena occur not only in oncogenesis but also, physiologically, during embryogenesis and are necessary for the maintenance of adult tissue homeostasis as well. We previously demonstrated that HGF and its receptor c-MET are expressed and active in the testis from early embryonic development to an adult stage [35], influencing many activities of testicular somatic and germ cells, both in humans and in rodents [35–38]. It is worth highlighting that, the most accepted theory about the onset of this kind of tumors states that the gonocyte block of differentiation is due to a combination of genetic and epigenetic aberrations with micro-environmental cues that jointly lead to the disease [39, 40]. This has led to coining a word, “genvironment”, which designates the close interaction between environmental factors, diffusible signals and gene expression regulation in the onset of TGCTs [41]. Intriguingly, in TGCT patients, an inverse correlation between progression-free survival and some circulating cytokines, including HGF, has been recently found [42]. In this respect, it is worth mentioning that c-MET availability has also been correlated with resistance to radio- and chemotherapy in different cancer types [43–45]. Altogether, these observations lead us to hypothesize that the deregulation of c-MET activation could represent one of the molecular mechanism responsible for the TGCT onset and/or progression.

Therefore, we have analyzed the expression pattern of the HGF/c-MET system and its possible role in pathogenesis of TGCTs. For this purpose, we used the seminoma cell line TCam-2, an intermediate-seminoma/non-seminoma cell line NCCIT and an embryonal carcinoma cell line NT2D1 as experimental models. We evaluated biological responses to HGF, such as proliferation, migration and invasion. Next, we studied the immunoreactivity of c-MET in histological samples of all major variants of TGCTs, aiming to correlate clinical data with the data provided by an *in vitro* study.

## RESULTS AND DISCUSSION

### TGCT cell lines have different copy numbers of *c-MET* gene

**Figure 1:**
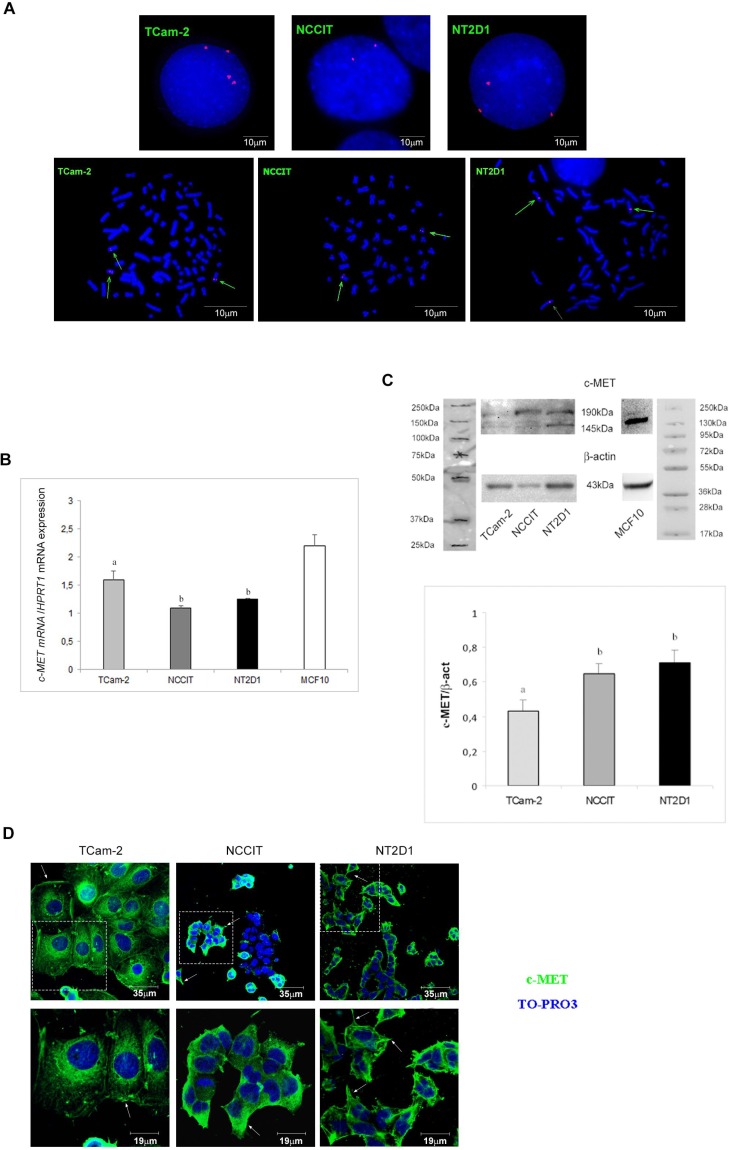
c-MET expression in (T)GCT cell lines (**A**) *c-MET* gene copy number in TCam-2, NCCIT and NT2D1 cell lines in interphasic nuclei and metaphases spread. Multiple copies of *c-MET* gene in TCam-2 and NT2D1 cells were detected. (**B**) *c-MET* qPCR in TCam-2, NCCIT and NT2D1 cell lines cultured in medium containing 10% FBS. A higher expression level in *c-MET* in TCam-2 respect to the other two cell lines was detected (b *vs* a *p* < 0.05). Quantitative sample value was normalized for the expression of *HPRT1*. MCF-10 cells were used as positive control (**C**) Western blot analysis of c-MET protein in TCam-2, NCCIT and NT2D1 cell lines cultured in medium containing 10% FBS. Two different bands, corresponding to the protein precursor (190 KDa band) and the mature full length β-chain (145 KDa band), were detected. Densitometric analysis of the 145 KDa bands, normalized versus β-actin, is reported (b *vs* a *p* < 0.05). MCF-10 cells were used as positive control. (**D**) Confocal microscopy analysis of c-MET subcellular distribution observed by immunofluorescence in TCam-2, NCCIT and NT2D1 cells cultured in medium containing 10% FBS. Representative images of the optical spatial series, withdrawn at nuclear level, have been reported. A plasma membrane signal (arrows) and a cytoplasmic diffused signal are detectable in all the cells. NCCIT cells show a double population: one c-MET-positive and the other c-MET-negative. FITC signal merged with TO-PRO3 staining is reported. The images in the upper panel are lower magnification (scale bar 35 µm), whereas the images in the lower panel are higher magnification of the field in the dotted square (scale bar19 µm). All experiments were performed at least in triplicate and reported as mean ± SEM.

As previously mentioned, the literature data report a gain of chromosome 7 in type II GCTs. Since *c-MET* gene is located in the ch7q31 region, we decided to use *fluorescence in situ hybridization* (FISH) analysis to assess if GCT cell lines gained the *c-MET* gene. As expected, the DAPI (4’,6-diamidino-2-phenylindole) images revealed the presence of global aneuploidy in all cell lines. Interphase and metaphase studies detected the presence of 2 signals of MET probe on two distinct chromosomes in NCCIT cells, and 3 signals on three distinct chromosomes in NT2D1 cells, whereas in TCam-2 cells 4 different signals were found, including 2 on two distinct chromosomes and 2 on a single derivative chromosome (Figure 1A). The latter observation is in line with the already demonstrated tetraploid karyotype of TCam-2 cells [46].

### TGCT cell lines differentially express c-MET receptor

The *c-MET* gene expression level has been assayed by real-time PCR analysis. We found that TCam-2, NCCIT and NT2D1 cells express *c-MET* at different levels. Particularly, in agreement with the reported FISH analyses, TCam-2 cells express higher levels of *c-MET* mRNA compared with NCCIT and NT2D1 cells (Figure 1B, Supplementary Figure 5). To evaluate c-MET protein expression, we performed both western blotting and immunofluorescence assays. By western blot analysis, we detected a higher expression of full-length c-MET protein (145 kDa) in NT2D1 cell line compared with NCCIT cells and, surprisingly, even with TCam-2 cells (Figure 1C, Supplementary Figure 5). This observation is a strong indication of a post-transcriptional regulation of c-MET protein biosynthesis, which reinforces the already demonstrated notion that these cancers are regulated at the post-genomic level [47].

Immunofluorescence analysis by confocal microscopy, revealed that in all cell lines, c-MET protein appears localized, as expected, at the plasma membrane level, since c-MET is a membrane receptor. However, besides the plasma membrane localization, a cytoplasmic diffused signal is also detectable. It is tempting to speculate that the latter c-MET localization indicates, the presence of the immature receptor form in the endoplasmic reticulum and/or its localization in endosomal vesicles containing the recycled receptor. In both cases, this observation would suggest that a basal turnover of c-MET protein is present in all TGCT cell lines, even if further investigations are needed to confirm this hypothesis. It is worth highlighting that we observed a dual population in NCCIT cell line: one c-MET-positive (approximately the 68% of the whole population) and the other c-MET-negative, which, reasonably, are differentially sensitive to receptor stimulation (Figure 1D).

### TGCT cell lines do not express and secrete HGF

**Figure 2:**
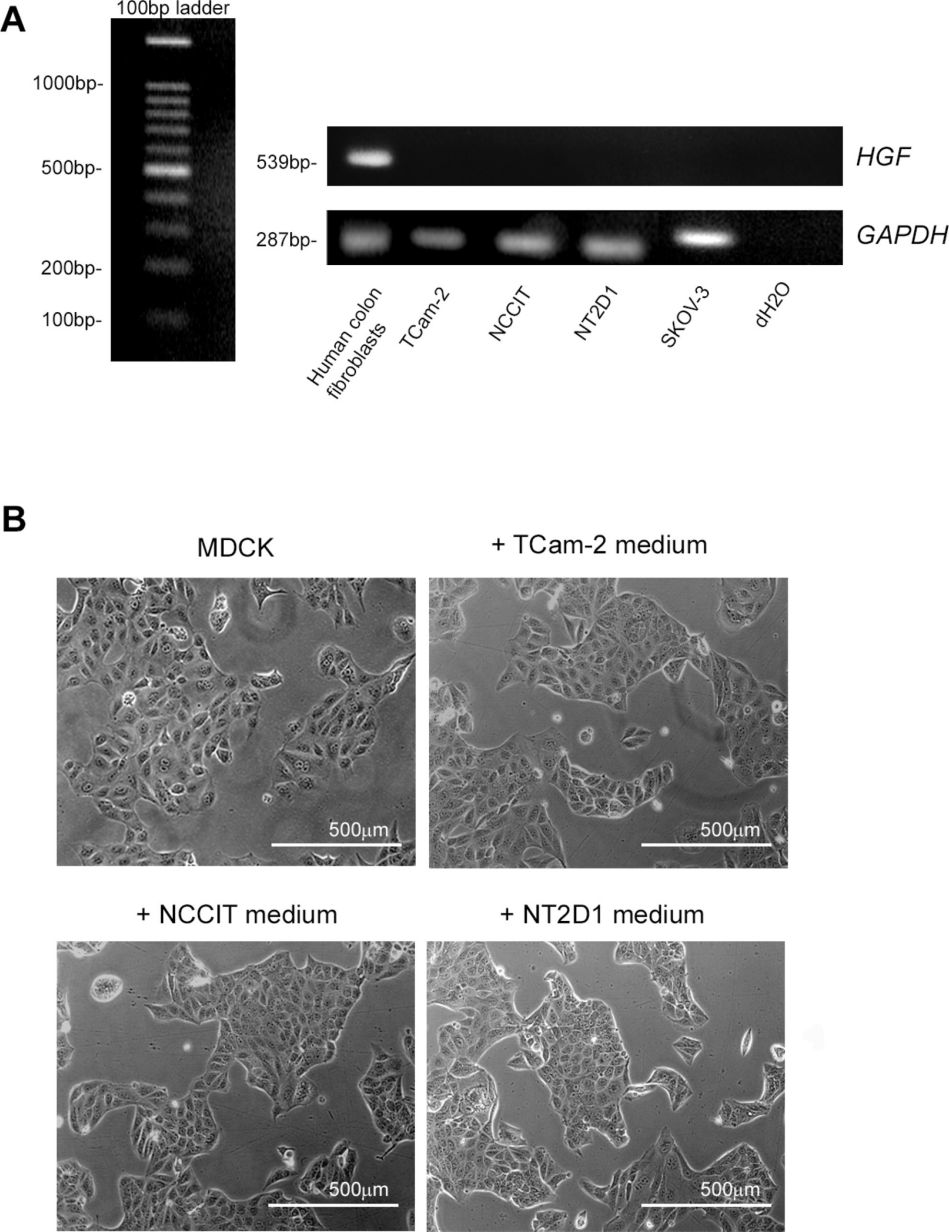
HGF expression in (T)GCT cell lines (**A**) RT-PCR of *HGF* expression in TCam-2, NCCIT and NT2D1 cell lines. No band for *HGF* cDNA amplicon was detected in all the cell lines. Human colon fibroblast-derived cDNA was used as positive control, whereas SKOV-3 cDNA and water alone were used as negative controls. The evaluation of *GAPDH* expression was used as loading control. (**B**) Scatter activity assay of TCam-2, NCCIT and NT2D1 conditioned media on MDCK colonies. Cluster morphology of un-stimulated MDCK or stimulated with conditioned media of TCam-2, NCCIT and NT2D1 were reported. No scatter activity was observed in MDCK cells stimulated with GCT cell line conditioned media. All experiments were performed at least in triplicate and reported as mean ± SEM. (scale bar: 500 µm).

To assess if our cell lines express *HGF* mRNA, we performed RT-PCR experiments, which revealed that TCam-2, NCCIT and NT2D1 cell lines do not express *HGF* mRNA. Human colon fibroblasts were used as a positive control and SKOV-3 cells were used as a negative control for the expression of this gene, as shown in Figure 2A. To investigate the availability of HGF protein in TGCT cell lines and to evaluate the possibility of a cellular storage and subsequent secretion of this molecule, we also performed a scatter activity assay, which tested the ability of cultured cell-conditioned media to induce scatter of MDCK cell clusters. Human colon fibroblast-conditioned medium (not shown) or HGF added medium were used as positive controls. The scatter phenotype in MDCK cells was observed with human colon fibroblasts (positive control, not shown) and recombinant HGF-added media (Supplementary Figure 1), while conditioned media obtained from SKOV-3 (negative control, not shown), TCam-2, NCCIT and NT2D1 cells did not show this activity (Figure 2B). To assess the specificity of the effect of c-MET inhibitor PF-04217903 on HGF-mediated activities, we treated MDCK cells with HGF+PF-04217903 and obtained total inhibition of the HGF-induced scatter activity. The scoring profile of the scatter activity of different doses of HGF and different doses of PF-04217903 is provided in Supplementary Figure 1.

The absence of HGF expression and secretion was expected due to the origin of these cellular lines. Considering that all these (T)GCT cell lines, grow as epithelial sheets, we evaluated the scatter activity of HGF on all (T)GCT cell lines, but observed no HGF-induced cell dispersion in any of the (T)GCT lines considered. The lack of HGF expression and secretion in these cells let us to use the HGF as a stimulating factor in different functional experiments, mimicking the testicular microenvironment in which this factor is commonly available.

### HGF administration increases NT2D1 proliferation, but does not affect TCam-2 and NCCIT cell growth

**Figure 3:**
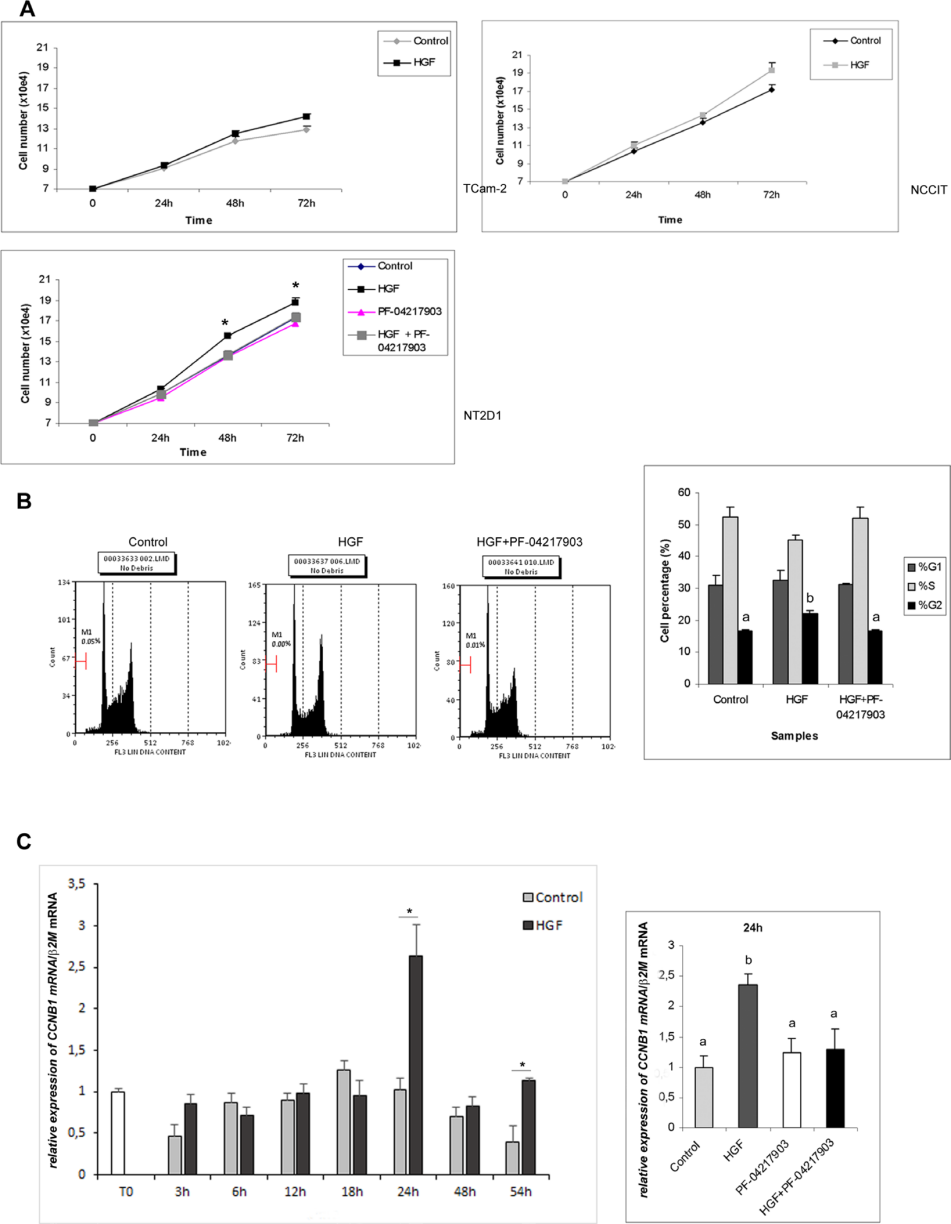
Proliferation assays after HGF treatment in TGCT cell lines (**A**) Cell count of TCam-2, NCCIT and NT2D1 cultured in medium containing 2% FCS with or without (Control condition) HGF treatment, for 24–48 and 72 h of culture. An increase in cell number in NT2D1 has been observed after 48 and 72 h of stimulation (^*^p < 0.05). C-MET inhibition by PF-04217903 abrogates the HGF induced cell number increase. No significant increase in cell number has been observed in the other cell lines at any culture time investigated. (**B**) Cell cycle FACS analysis on NT2D1 cells in Control condition and after 48 h of HGF stimulation. A significant increase of cells in G2/M phase (%) in HGF stimulated samples has been reported (b vs a *p* < 0.05 *p* < 0.05). c-MET inhibition by PF-04217903 abrogates the HGF-induced effect. In the left part of the panel the graphical representation of cell cycle profile is reported. In the right part the graphical representation of cell percentage in the different phases of cell cycle is shown. (**C**) Left part of the panel: time course analysis followed by real-time PCR of *CCNB1* gene on NT2D1 cell after HGF administration. A significant increase in *CCNB1* gene expression level is observable after 24 and 54 h of stimulation (^*^p < 0.05). Right part of the panel: real-time PCR of *CCNB1* gene in control condition and after HGF, PF-04217903, HGF+PF-04217903 stimulation on 24 h cultured NT2D1. PF-04217903 treatment abrogates the HGF induced effect. All experiments were performed at least in triplicate and reported as mean ± SEM.

In the light of the results reported above, we evaluated different aspects of HGF activity on (T)GCT cells, starting from the well-known capability of this factor to promote cell proliferation. We cultured cells in the absence and in the presence of HGF for 24, 48 and 72 h. As shown in Figure 3A, HGF administration does not significantly modify the numbers of TCam-2 and NCCIT cells compared with the control samples, at all analyzed times, even though in NCCIT cells we observed a positive trend after HGF stimulation. In NT2D1 cells, HGF promotes cell proliferation starting from 24 h of culture. This increase becomes statistically significant after 48 and 72 h. To assess the specificity of HGF activity on proliferation, we treated NT2D1 cells with the c-MET selective inhibitor PF-04217903 under the same experimental conditions. The PF-04217903 completely abrogates the HGF-induced effect on cell proliferation both 48 and 72 h of culture (Figure 3A, Supplementary Figure 5). PF-04217903 does not exert any effect on cell viability (dead cells: 3,57% ± 0,08 *vs* 3,67% ± 0,29 control *vs* PF-04217903 respectively). To further investigate the HGF effect on NT2D1 cell proliferation, we performed a cell cycle FACS-based analysis. Cell starvation let us synchronize NT2D1 cellular cycle (68,8% ± 0,6 cells in G1 phase after 16 h starvation). After 48 h of HGF administration, that is, the culture time at which the cell number increase becomes statistically significant, we found a higher percentage of cells in G2/M phase committed to mitosis compared with the control samples (Figure 3B). The co-administration of HGF and c-MET inhibitor reverts the increased percentage of cells in G2/M phase induced by HGF, demonstrating the specificity of this response (Figure 3B, Supplementary Figure 5). For a deeper exploration of the molecular mechanism of this biological process activated by HGF in NT2D1 cell line, we analyzed the expression level of the cyclin B gene (*CCNB1*), a key regulator of G2/M transition. We performed a time-course experiment followed by real time PCR assay and found that HGF up-regulates *CCNB1* gene starting from 24 h of culture (Figure 3C, Supplementary Figure 5). At the same culture time, we treated cells with PF-04217903 alone and with HGF+PF-04217903. As shown in Figure 3C, we observed that in HGF+PF-0421790-treated cells *CCNB1* expression is comparable to the control level. The *CCNB1* gene up-regulation after 24 h of HGF administration indicates that the HGF action occurs early, but that the duration of the phases of the cycle is prolonged due to the low FBS concentration (2%) at which these experiments were performed. This is the probable reason why we observed a significant difference in the numbers of cells only after 48 h of culture and not at earlier culture times. Since *CCNB1* gene up-regulation is a cyclic event, we prolonged the time-course experiments and obtained another peak of *CCNB1* mRNA, although less sharp, at 54 h post-HGF administration (Figure 3C). This observation let us conclude that the HGF is still able to promote cell proliferation in NT2D1 cells even at this culture time. This result is in line with the reported maintenance of the cell number increase 72 h after HGF administration.

### HGF acts as chemo-attractant for NCCIT and NT2D1 cells

**Figure 4:**
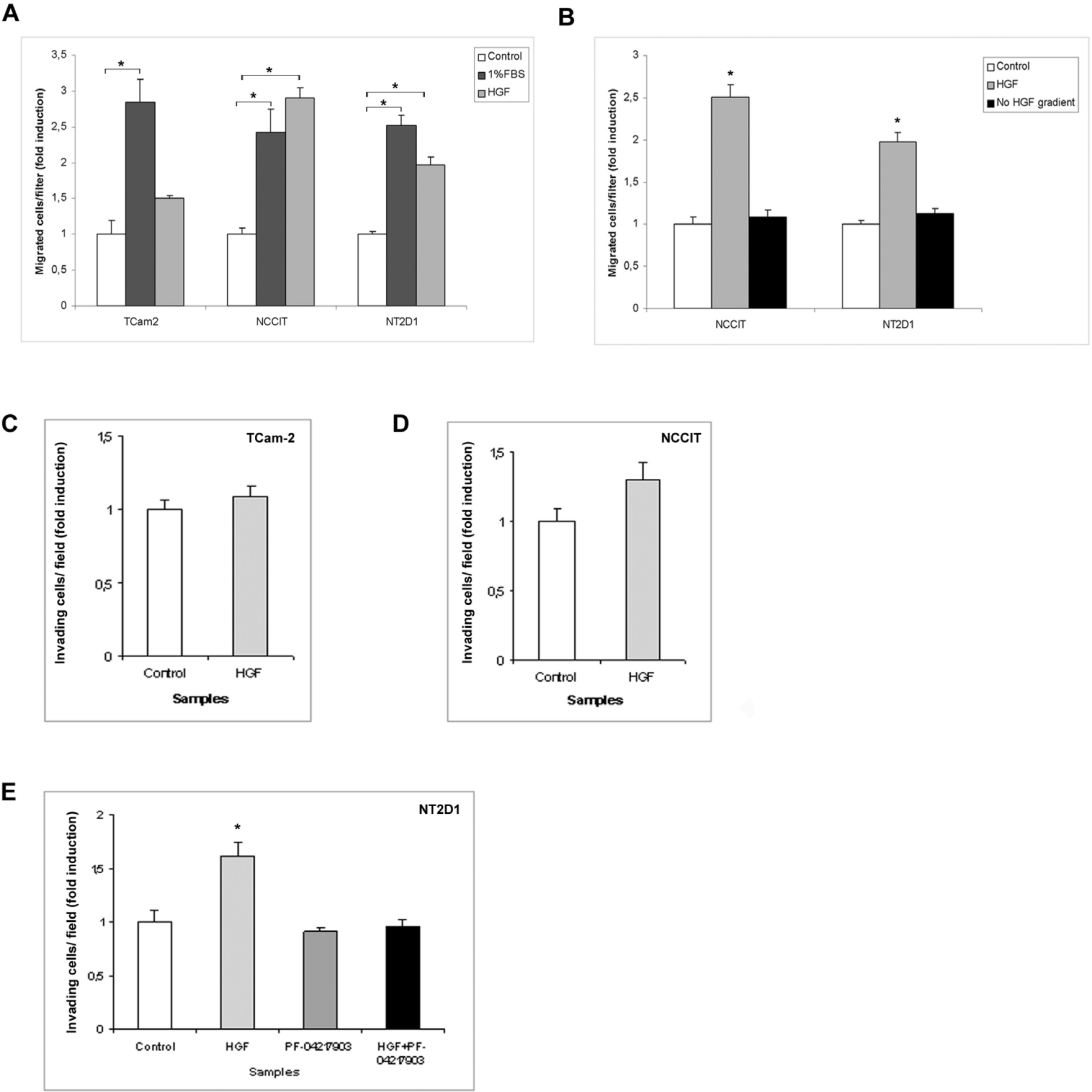
Boyden chamber and Matrigel invasion assays after HGF treatment in (T)GCT cell lines (**A**) Chemotactic activity of HGF on TCam-2, NCCIT and NT2D1 after 5 h of chemo-attraction. A significant increase in polarized migration has been observed, compared with the negative control (serum free medium + 0.1% BSA), in NCCIT and NT2D1 cell lines (^*^p < 0.05) whereas no modulation in TCam-2 was observed. 1% FBS was used as chemo-attractant positive control. (**B**) Abrogation of HGF gradient prevents the polarized migration in NCCIT and NT2D1 cells. TCam-2 (**C**), NCCIT (**D**) and NT2D1 (**E**) invasion assay in control condition (medium + 2% FBS + 0.1%BSA) and after 24 h of HGF stimulation. No significant increase has been observed in (C) and (D), whereas a significant increase has been reported in (E) (^*^p < 0.05). c-MET inhibition by PF-04217903 abrogates the HGF-induced increase of invasion in NT2D1 cell line. All experiments were performed at least in triplicate and reported as mean ± SEM.

To better characterize the role of *c-MET* proto-oncogene in seminoma and non-seminoma cancer progression, we investigated the capability of HGF to act as a chemo-attractant on the TGCT cell lines. We performed a Boyden chamber migration assay using HGF molecule in the lower chamber. Our results demonstrate that HGF does not significantly modify cell migration in TCam-2 cells. In contrast, HGF increases cell migration in NCCIT and NT2D1 cell lines (Figure 4A, Supplementary Figures 2 and 6). To abrogate the molecular gradient, HGF was added to both the upper and lower chambers. In this experimental condition, NCCIT and NT2D1 cell migration is comparable to the control (Figure 4B, Supplementary Figures 2, 6). Our data confirm that HGF acts as a chemo-attractant for these cell lines. Since HGF is mainly expressed by interstitial/connective cells, its chemo-tactic activity on these tumor cell lines is relevant and indicates a potential HGF role in the chemo-attraction of these cells in the interstitial compartment during their escape from the tubular compartment, i.e., the transition from to GCNIS to invasive growth.

### c-MET activation increases NT2D1 cell capability of invasion

**Figure 5:**
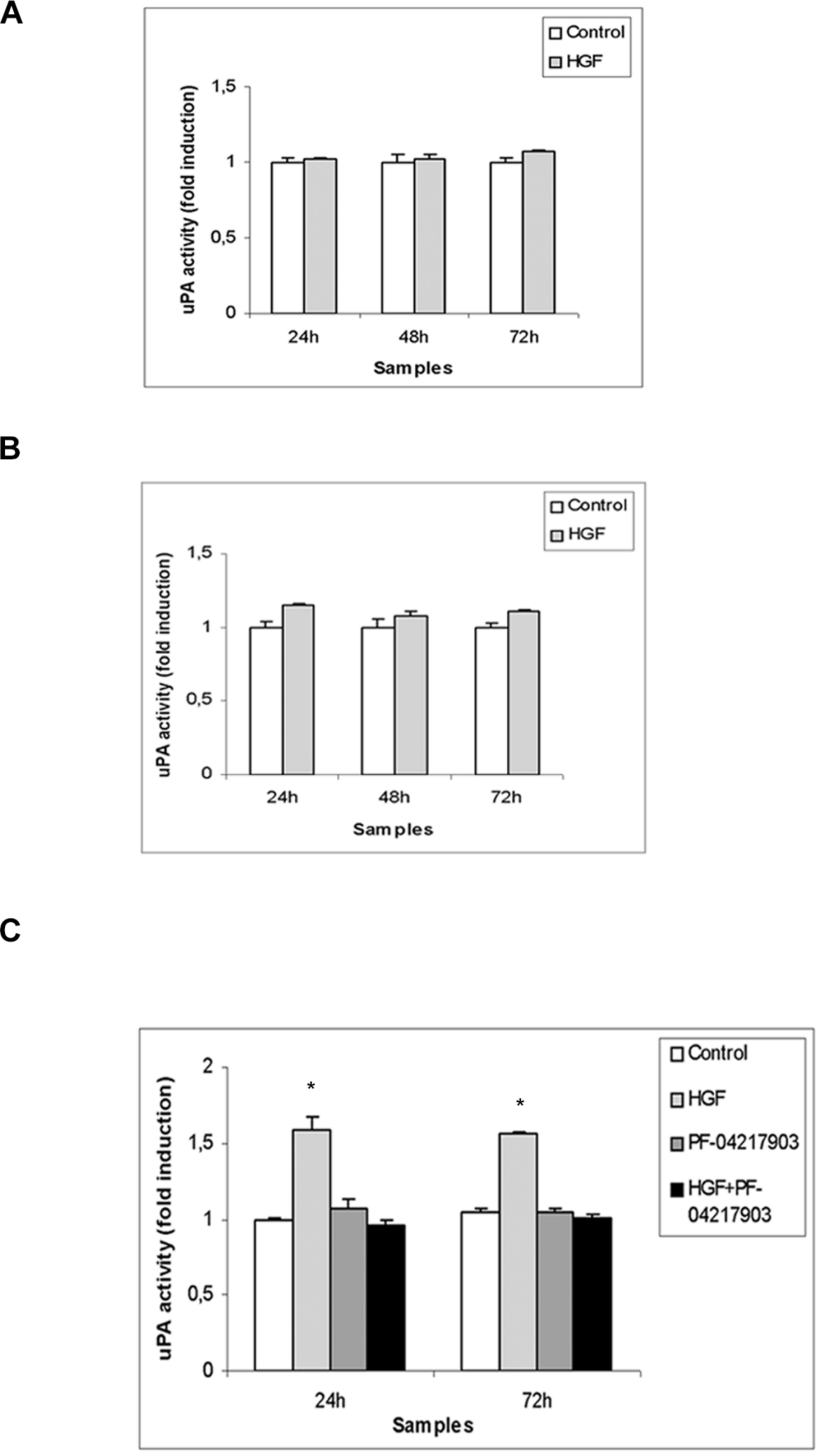
uPA activity in (T)GCT cell line conditioned media after HGF stimulation Densitometric analysis of casein-gel zymographies of TCam-2 (**A**), NCCIT (**B**) and NT2D1 (**C**) conditioned media, evaluating uPA activity in control condition (medium containing 2% FBS) and after 24–48–72 h of HGF stimulation. No significant increase has been observed in (A) and (B) in uPA activity, at all the analysed culture times. A significant increase has been reported in uPA activity on NT2D1 cell line (C) (^*^p < 0.05). C-MET inhibition by PF-04217903 abrogates the HGF-induced increase of uPA activity in NT2D1 cells. All experiments were performed at least in triplicate and reported as mean ± SEM.

**Figure 6:**
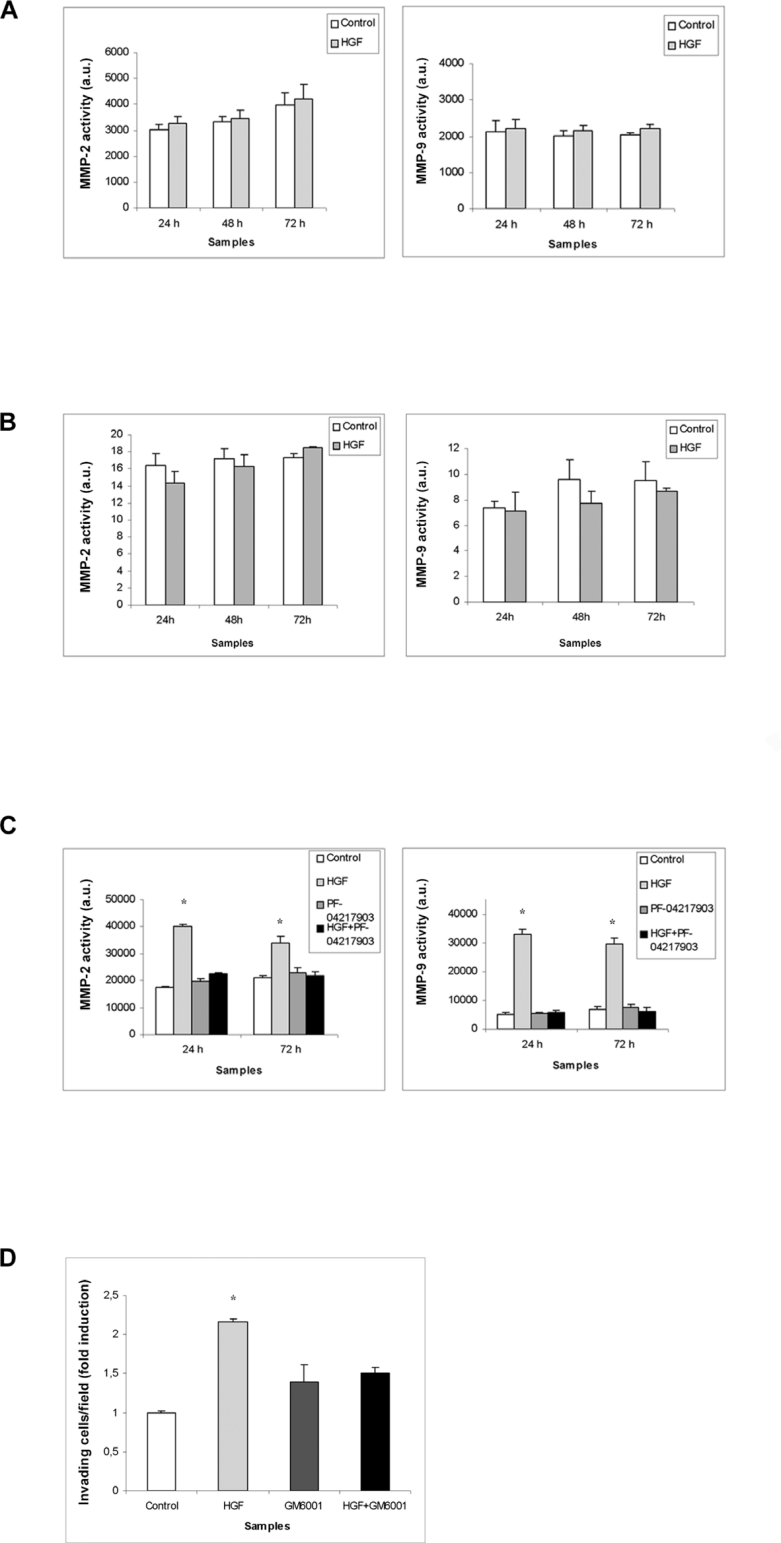
MMPs activity in (T)GCT cell line conditioned media after HGF stimulation Densitometric analysis of gelatin-gel zymographies of TCam-2 (**A**), NCCIT (**B**) and NT2D1 (**C**) conditioned media evaluating MMP activity in control condition (medium containing 2% FBS) and after 24-48-72 h of HGF stimulation. No significant increase has been observed in (A) and (B) in MMP activity, at all the analysed culture times. A significant increase has been reported in MMP activity on NT2D1 cell line (C) (^*^p < 0.05). C-MET inhibition by PF-04217903 abrogates the HGF increase of MMP activity in NT2D1 cells. (**D**) Effect of MMP inhibition on NT2D1 Matrigel invasion assay. MMP inhibition abrogates HGF-dependent invasive behaviour of NT2D1 cells. All experiments were performed at least in triplicate and reported as mean ± SEM.

We decided to investigate another aspect of tumorigenesis such as cell invasion. We performed an invasion assay using Matrigel basement-coated chambers. Cells were cultured on Matrigel layer and treated with HGF, PF-04217903 or both factors for 24 h. We found that HGF treatment significantly modulates the capability of invasion of NT2D1 cells (Figure 4E), whereas in TCam-2 (Figure 4C) and NCCIT (Figure 4D) cells HGF treatment does not modify cell behavior in terms of invasive potential (Supplementary Figure 3). Experiments performed to verify the specificity of this response in NT2D1 cells with the co-administration of HGF+PF-04217903 show a reversion of the HGF-induced effect. PF-04217903 alone does not modify the invasive capability of NT2D1 cells (Figure 4E, Supplementary Figure 3). Next, we tested the modulation of proteolytic enzymes of extracellular matrix, such as urokinase plasminogen activator (uPA) (Figure 5A–5C) and metalloproteinases 2 and 9 (MMP-2/MMP-9) (Figure 6A–6C) in cell conditioned-media. The uPA activity at 24 h and 72 h of culture increases with HGF treatment only in NT2D1 cells (Figure 5, Supplementary Figure 4). Additionally, MMP-2 and MMP-9 activities are increased in this cell line (Figure 6C, Supplementary Figure 4). Treatment with HGF+PF-04217903 in NT2D1 cells reverted the HGF effect on uPA and MMP-2/MMP-9 activity (Figures 5–6, Supplementary Figures 4, and 6). Based on these results, we performed the Matrigel invasion assay in NT2D1 cells using a MMPs inhibitor, GM6001, for the same culture time. We observed that the co-administration of HGF with GM6001 reverted the HGF effect on cell invasion (Figure 6D), thus confirming that HGF-induced invasive potential is metalloprotease-dependent (Supplementary Figure 3).

### HGF administration modulates c-MET activity, expression and availability in NT2D1 cells

**Figure 7:**
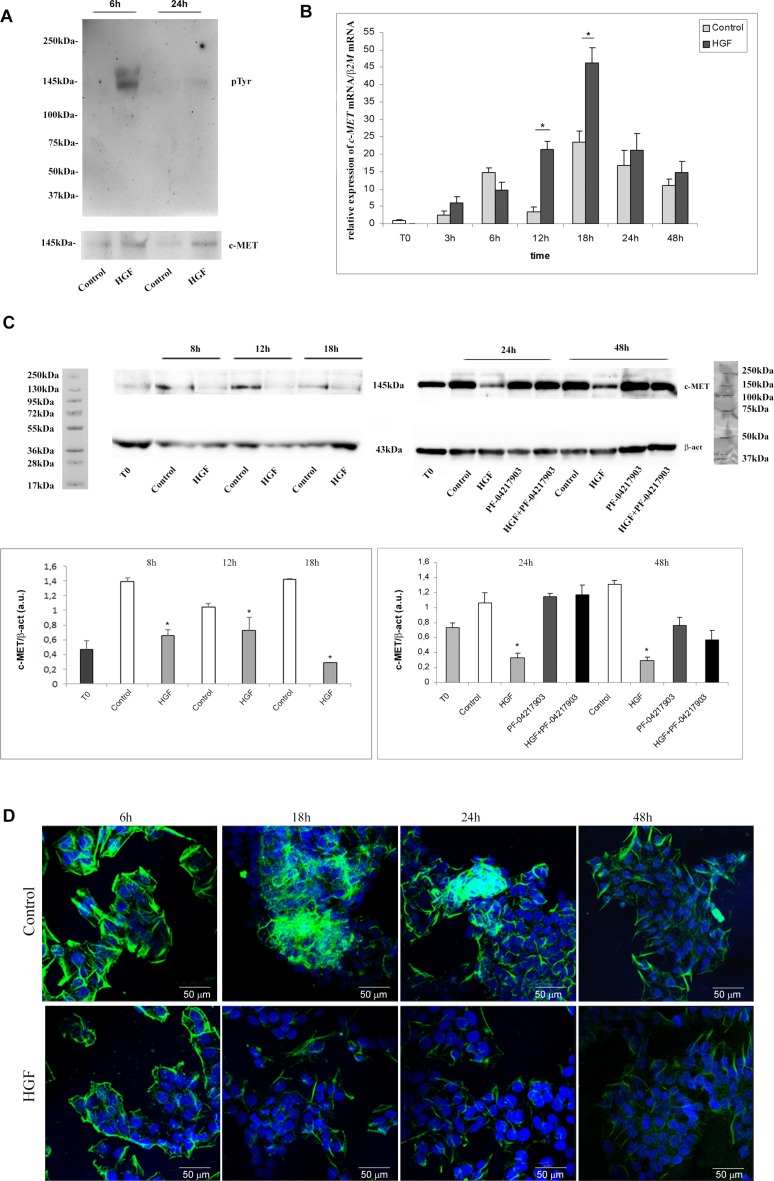
c-MET phosphorylation, expression, availability and distribution pattern in NT2D1 cells after HGF stimulation (**A**) Immunoprecipitation of c-MET protein followed by p-Tyr blot in Control condition (2%FBS medium)and after 6 h and 24 h HGF stimulated NT2D1. c-MET receptor phosphorylation has been observed after 6 h stimulation, whereas no phosphorylation is reportable after 24 h. (**B**) Time-course analysis followed by real-time PCR of c-Met gene on NT2D1 cell after HGF administration. A significant increase in c-Met gene expression level is observable after 12–18 h of stimulation (^*^p < 0.05). (**C**) Western blot analysis of c-MET protein at time 0 of culture, as well as in control condition and 8, 12, 18, 24 and 48 h after HGF stimulation. A decrease of c-MET bioavailability has been detected in all the culture times considered (^*^p < 0.05). Upper part of the panel: Western blotting detected bands are reported. Lower part of the panel: densitometric analysis of the bands, normalized versus β-actin, are reported. (**D**) c-MET immunofluorescence in control condition and after 6, 18, 24 and 48 h after HGF stimulation. All experiments were performed at least in triplicate and reported as mean ± SEM (scale bar: 50 µM).

It is well known that c-MET signaling cascade starts with phosphorylation events in the intracellular domain of the receptor. Since we observed biological effects upon HGF stimulation mainly in NT2D1 cell line, we evaluated the phosphorylation rate of c-MET protein by performing c-MET immunoprecipitation followed by p-Tyr blot only in this cell line. As shown in Figure 7A, after 6 h of treatment we observed receptor phosphorylation in HGF-stimulated samples, whereas after 24 h no more c-MET phosphorylation was observed (Figure 7A). Next, we investigated if HGF stimulation could modulate *c-MET* expression. Cells were treated with HGF for different periods of time (3, 6, 12, 18, 24, and 48 h). After 12 and 18 h, by qRT-PCR assay, we observed an up-regulation of the *c-MET* gene expression level in HGF-treated samples compared with the control cells (Figure 7B, Supplementary Figure 5). Surprisingly, western blot analysis of full-length c-MET protein showed a decrease in HGF-stimulated samples, compared with the control, at all culture times considered (Figure 7C, Supplementary Figure 5). The results obtained by western blot analysis were confirmed by immunofluorescence experiments (Figure 7D), in which a decrease of c-MET membrane positive signal after HGF administration is evident, even though c-MET receptor never disappears from the plasma membrane of these cells. These apparently conflicting results, could be explained by the well-known c-MET pathway activation, which triggers the turnover of c-MET protein as a consequence of the ligand-mediated endocytosis [48–50]. According to this hypothesis, the up-regulation of *c-MET* mRNA observed at 12 and 18 h after HGF administration could represent a mechanism of compensation for the turnover of this membrane receptor. Alternatively, it could be hypothesized that the increase in c-MET mRNA levels is translated by NT2D1 cells into c-MET soluble isoforms or some truncated isoforms that the western blot and immunofluorescence analyses cannot reveal under these experimental conditions. Further investigations are needed to clarify this point.

We treated cells with c-MET inhibitor PF-04217903 alone or with HGF for the same culture times; c-MET inhibitor does not affect protein expression, but the co-administration of HGF+PF-04217903 reverts the HGF-dependent effect, and c-MET protein level appears comparable with the control samples (Figure 7C, Supplementary Figure 5).

### C-MET immunoreactivity in TGCT samples

The c-MET immunoreactivity in 150 tissue samples from the different histological components (GCNIS, SE, EC, YST, CHC and TE) was studied, using c-MET immunohistochemistry (IHC) on tissue microarrays (TMAs). The staining was scored, and the results are shown in Table 1. GCNIS has membranous staining for c-MET, in some cases combined with weak nuclear staining; cytoplasmic staining is not observed. Seminoma has a weaker membranous staining, a weak diffuse cytoplasmic staining pattern and stain-negative nuclei. It is worth mentioning that GCNIS and SE cells contain a large amount of glycogen, which pushes cellular components to the periphery of the cell. This phenomenon may explain the lack of visible cytoplasmic staining in SE and GCNIS cells. Moreover, the Sertoli cells surrounding the GCNIS cells also have membranous staining for c-MET, making it difficult to assess the contribution of the two cell types to the membranous staining of GCNIS cells in the spermatogonial niche.

**Table 1:** Summary results of c-MET immunoreactivity scoring analyses in TGCT histological samples

Tumor component	M	C	N
GCNIS	++	−	+
SE	+	+	−
EC	++	++	−
YST (E) (NE)	+++ ++	+++ ++	− −
CHC (C) (S)	+++ −	++ +	− −
TE	+++	++	−

c-MET staining intensities were evaluated separately for nuclear, cytoplasmic and membranous immunoreactivity on a scale from - to +++.

M: membranous staining; C: cytoplasmic staining; N: nuclear staining; E: epithelial component; NE: non-epithelial component; C: cytotrophoblast; S: syncytiotrophoblast.

**Figure 8:**
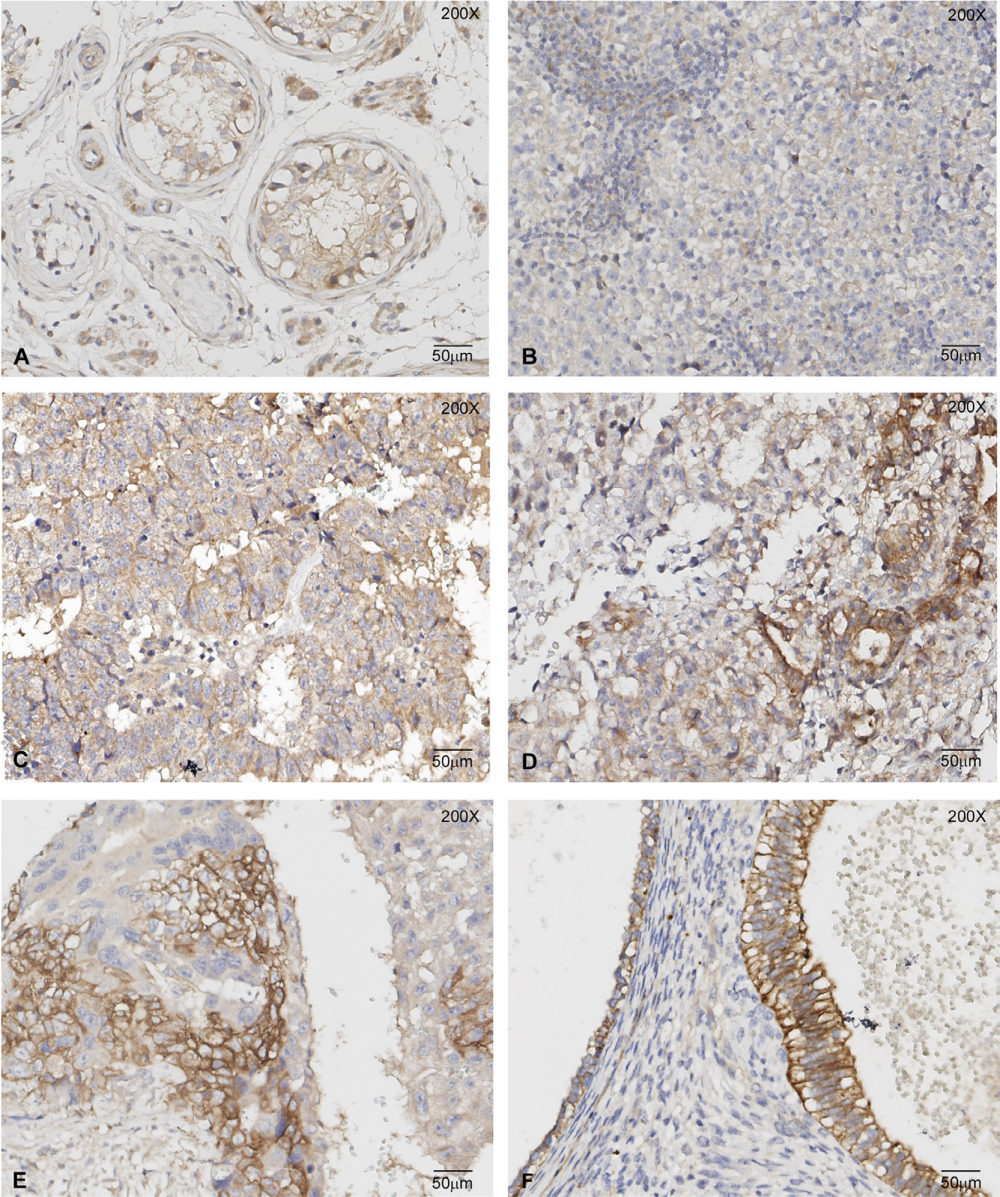
c-MET immunoreactivity in TGCT histological samples Representative images of c-MET immunohistochemistry on GCNIS (**A**), Seminoma (**B**), Embryonal Carcinoma (**C**), Yolk Sac Tumour (**D**), Choriocarcinoma (**E**) and Teratoma (**F**) samples. The highest c-MET immunoreactivity was observed in the epithelial components of Yolk Sac Tumour, Choriocarcinoma and Teratoma.

Embryonal carcinoma cells have stronger membranous and cytoplasmic staining than seminoma and GCNIS, particularly in necrotic areas and at the luminal surface of tubular structures of embryonal carcinoma. The nuclei of embryonal carcinoma are stain-negative. The strongest expression of c-MET was found in teratoma (all epithelial components), in epithelial components of yolk sac tumor, and in the cytotrophoblastic component of choriocarcinoma. These components show strong membranous and cytoplasmic staining. The non-epithelial component of yolk sac tumor shows weak membranous and cytoplasmic staining; syncytiotrophoblast only has a weak cytoplasmic expression (Figure 8). Next, we assessed if c-MET immunoreactivity observed in TMA section was representative of the whole-tissue sections of these tumors. As expected, in whole-tissue sections, we observed a more heterogeneous pattern, but the c-MET distribution was essentially similar to that seen in the TMA. The impact of the presence of c-MET on the origin and further progression of (T)GCTs, based on these results, remains to be determined, although some conclusions can be drawn. The transition from a solely membranous localization to a combined membranous and cytoplasmic staining in seminoma and various non-seminomatous elements is intriguing. The unique nuclear staining in the GCNIS cells is of interest as well, although it is unclarified so far. It is clear that the possible histological diversity of (T)GCTs is not directly reflected by a differential staining pattern for c-MET. This diversity is at least associated with the lineage of differentiation, which is demonstrated by the membranous presence in the cytotrophoblast of choriocarcinoma and the absence in the syncytiotrophoblast component.

### Concluding remarks

Herein, we demonstrate that:

- *c-MET* gene appears duplicated in TCam-2 (four copies) and NT2D1 (three copies) cells, as part of a likely whole-genome duplication, whereas NCCIT cells show a normal *c-MET* gene copy number, due to a possible gene loss. The *c-MET* mRNA level appears consistent with the described genetic alterations. However, the c-MET full-length protein content appears to be differently regulated in these (T)GCT cells, being more abundant in NT2D1 cells and less abundant in TCam-2 cells.

- The (T)GCT cell line that expresses highest levels of full length c-MET protein, is also the most sensitive to HGF administration, as expected. In particular, we observed that NT2D1 cells react to HGF by increasing their proliferation, polarized migration, and invasion trend, whereas TCam-2 cells do not seem to significantly respond to HGF, as far as the analyzed parameters are concerned. Interestingly, NCCIT cells appear capable of migrating in a polarized manner after HGF stimulation, whereas the proliferation and invasion assays failed to show a statistically significant modulation, although there was a positive trend. It is worth highlighting, that our immunofluorescence analyses provide evidence that the NCCIT cell line consist of a mixed population of c-MET-positive and c-MET-negative cells. It is therefore conceivable that only a fraction of these cells could respond to HGF.

- Since NT2D1 appeared to be the most responsive cell line, we studied c-MET phosphorylation, and the regulation of its expression after HGF treatment. As expected, short-term exposure to HGF resulted in tyrosine phosphorylation of the full-length c-MET receptor, which is a prerequisite of the c-MET pathway activation. Interestingly, after HGF exposure, NT2D1 cells showed a lesser bioavailability of the c-MET receptor, strongly indicating that the activation of the c-MET pathway triggers the turnover of c-MET protein, due to the ligand-mediated endocytosis [48–50]. According to this hypothesis, a transient up-regulation of *c-MET* mRNA, occurs to compensate for the turnover of this membrane receptor.

- In line with the *in vitro* results, immunohistochemical c-MET analysis of GCNIS and seminoma lesions, showed a clear but weak membranous immunoreactivity. Embryonal carcinoma cells have stronger, mainly membranous staining. Notably, in all EC-derived (i.e., differentiated) components (choriocarcinoma, yolk sac tumor and teratoma) the highest c-MET immunoreactivity was found in epithelial components, irrespective of their aggressiveness, i.e., invasive behavior. A possible role of c-MET up-regulation in malignant behavior of TGCT, suggested by our *in vitro* findings, requires further investigation.

In conclusion, the results reported herein shed new light on TGCT biology opening new perspectives for a deeper investigation of the HGF/c-MET system in these pathologies, possibly allowing the identification of patients at low/high risk of progression. Additionally, the presence of c-MET protein in these cancer lesions could represent a novel target for multimodal therapies and even more personalized medicine.

## MATERIALS AND METHODS

### Cell culture

TCam-2 seminoma cells [46, 51, 52], kindly provided by Prof. Claudio Sette (in 2009), were cultured in RPMI 1640 (Sigma-Aldrich, cat. R8758) supplemented with Fetal Bovine Serum (FBS Gibco, cat. 10270) and penicillin/streptomycin (Sigma-Aldrich, cat. P0781). NCCIT (of a primary mediastinal Type II GCT origin) and NT2D1 cell lines were purchased from the ATCC (in 2015) and cultured in DMEM (Sigma-Aldrich, cat. D6546), supplemented with FBS, L-glutamine (Sigma-Aldrich, cat. G7513) and penicillin/streptomycin. SKOV-3 and MCF-10 cell lines were cultured in RPMI and DMEM respectively, supplemented with 10% FBS, L-glutamine and penicillin/streptomycin. All cell lines were used from 15 to 35 passages from the first establishment of the cell lines.

The treated cells received 40 ng/ml of HGF (human recombinant HGF; R&D Systems, cat. 294-HG), 50 µM of c-MET selective inhibitor PF-04217903 (Sigma-Aldrich, cat. SML0263), or 50 µM MMP inhibitor GM6001 (Calbiochem/Millipore, cat. 364205). The PF-04217903 toxicity was evaluated by trypan blue exclusion test. We tested different concentrations of PF-04217903 (5-10-20-50 µM) as suggested in the literature (Zou *et al.*, 2012). MMP inhibitor GM6001 was used as previously described in Ferranti *et al.*, 2012.

Primary human colon fibroblasts (HF), kindly provided by Dr. Alessandra Cucina (in 2015), and MDCK cells, purchased from the ATCC (in 1998), were cultured in DMEM supplemented with 10% FBS, L-glutamine (Sigma-Aldrich, cat. G7513)and penicillin/streptomycin and used from 5 to 10 passages. Mycoplasma testing was routinely done with the N-GARDE Mycoplasma PCR Reagent set (EuroClone, cat. EMK090020). The last mycoplasma testing was done in July 2017.

### FISH analysis

Cells cultured in medium+ 10% FBS were trypsinized and harvested for the fluorescence *in situ* hybridization (FISH) experiment according to standard cytogenetic procedures. FISH analyses were performed on interphase nuclei and metaphase spreads using the MET locus-specific identifier (LSI) specific probe (Abbott Molecular/Vysis, cat. 06N05-020) according to manufacturer’s instructions, and the cells were counterstained with DAPI solution. A minimum of 10 metaphase spreads and 50 nuclei were analyzed for each cell line using a fluorescence microscope (Nikon Eclipse E600). Image capture and processing were performed using the Genikon software (Nikon).

### RNA isolation, RT-PCR and qRT-PCR analyses

Cells cultured in medium+ 10% FBS were harvested and RNA was extracted using phenol-chloroform (TriReagent, Sigma-Aldrich, cat. T-9424). Two micrograms of RNA were reverse-transcribed using the M-MLV Reverse Transcriptase kit (Invitrogen, cat. 28025-013). The semi-quantitative PCR analysis for *HGF* (Fw primer: TCCATGATAGCCACACGAACAC; Rv primer: AGCGTACCTCTGGATTGCTT) was carried out in a reaction mixture containing 1 unit of Taq DNA polymerase (Genedirex, cat. MB101-0500). The cycle parameters for *HGF* were as follows: 45 sec at 94° C, 45 sec at 61° C, and 1 min at 72° C for 25 cycles. HF cDNA was used as positive control and *GAPDH* (Fw: CTTTTGCGTCGCCAG; Rv: TTGATGGCAACAATATCCAC) was used as a loading control. The 100 bp DNA ladder (GeneDirex, cat. DM001-R500) was used as molecular weight marker for amplicons loaded onto agarose gels.

Real-time PCR was performed using the ABI PRISM 7500 SDS (Life Technologies-Applied Biosystems, USA), the FluoCycle II^™^ SYBR^®^ Master Mix 2X (EuroClone, cat. ERD002250BIM) (thermal profile: 5 min at 95° C for 1 cycle, 15 sec at 95° C for 45 cycles, 60 sec at 60° C for fluorescent acquisition) and pre-designed SYBR Green Primers (Sigma-Aldrich) for *c-MET* (Fw primer: AGACACATTTCAATTGGTGG; Rv primer: GTAACTGAAGATGCTTGTCTC) and *CCNB1* genes (Fw primer: GACACCAACTCTACAACATTAC; Rv primer: GTCCTTGATTTACCATGACTAC). Quantitative sample values were normalized to the expression of *HPRT1*mRNA(Fw primer: TGCAGACTTTGCTTTCCTTGGTCAGG; Rv primer: 5′-CCAACACTTCGTGGGGTCCTTTTCA) and *β2M* (Fw primer: AAGGACTGGTCTTTCTATCTC; Rv primer: GATCCCACTTAACTATCTTGG). The relative level for each gene was calculated using the 2−ΔΔCt method [53] and the standard curve method.

### Protein isolation, immunoprecipitation and western blotting assays

Cells were homogenized in RIPA buffer (Sigma-Aldrich, cat. 0278) containing inhibitors of proteases and phosphatases (Roche, cat. 04693124001 and 04906837001 respectively). The protein content was determined using the BCA protein assay (Pierce, cat. 23221). For immunoprecipitation experiments, 250 µg of protein extracts was pre-cleared with the True Blot Anti-Rabbit Ig IP Beads (Rockland, cat. 00-8800-25) and then rotated overnight. at 4° C with rabbit anti-c-MET antibody (Cell Signaling Technology, 1:50, cat. 4560). lmmunocomplexes were collected using the True Blot Anti-Rabbit Ig IP Beads after rotation for 5 h at 4° C.

For western blotting assay, proteins and IP immunocomplexes were re-suspended in boiling Laemmli buffer under reducing conditions and then separated on 7% SDS–PAGE. The proteins were electro-transferred to nitrocellulose membrane (Protran, cod. 10 401 196). Non-specific antibody bindings was blocked by incubation with 5% BSA (Sigma-Aldrich, cat. A2153) in TBS-T buffer (20 mMTris, pH 7,6, 150 mM NaCl/0,1%Tween-20). Then, the membranes were incubated with rabbit anti-c-MET (Cell Signaling Technology, 1:500, cat. 4560), rabbit anti-c-MET D1C2 (Cell Signaling, 1:2000, cat.8198), mouse anti-pTyr clone 4G10 (Millipore, 1:500, cat. 05-321), mouse anti-β-actin (1:1000, Sigma-Aldrich, cat. A5441), and mouse anti-α-tubulin (Sigma-Aldrich, 1:1000, cat. T5168) antibodies for 16 hours at 4° C, followed by incubation with the appropriate HRP- (Amersham Bioscience, 1:3000, cat. NA9340V) or AP-conjugated secondary antibody (Sigma-Aldrich 1:3000, cat. A-4312). Immunocomplexes were detected using western blot chemiluminescent reagents (ECL western blotting detection reagent, Euroclone, cat. EMP011005 or CDP-star, PerkinElmer, cat. NEL602001KT) following the manufacturer’s instructions. The membrane images were acquired and analyzed by the ChemiDoc XRS with Image Lab software (Bio-Rad Laboratories). The Page Ruler Plus Prestained Protein Ladder and Precision Plus Protein All Blue Standards (Bio-Rad Laboratories) have been used as molecular weight markers.

### Immunofluorescence analysis

Cells cultured for 24 h in 10% FBS (Figure 1), or at different culture times in 2% FBS with or without HGF, on ibidi slides (µ-Slide 8 well, ibidi, cat. 80826) were fixed in 4%paraformaldehyde in PBS (pH 7.4) at 4° C for 10 min. Then, cells were permeabilized in PBS/1% BSA/0.1% Triton for 3 h, and incubated overnight with mouse anti-c-MET antibody (mouse monoclonal, Novocastra, 1:50, cat. NCL-cMET). Cells were incubated for 1 hour and 30 minutes with a FITC-conjugated goat anti-mouse IgE (Santa Cruz, 1:80, cat. sc-2082). TO-PRO3 iodide fluorescent dye 642/661 (1:5000 in PBS, Invitrogen, cat. T3605) was used for nuclei staining. As a negative control, the primary antibody was omitted. Immunofluorescence experiments were analyzed under a Leica Confocal Microscope (Laser Scanning TCS SP2 equipped with Kr/Ar and He/Ne lasers) by performing optical spatial series with a step size of 2 µm.

### Scatter activity assay

The scatter activity of TGCT cell culture media, obtained after 72 h incubation, was measured on colonies of MDCK cells, as previously described [54]. As positive controls, HGF- (10 to 60 ng/ml) or human colon fibroblast-conditioned media (not shown) were added. The HGF concentration of 40 ng/ml is the lowest that triggers the 100% of scatter activity of MDCK cells. To test the specificity of HGF action, PF-04217903 (5–10–20–50 µM) was used. The concentration of 50 µM inhibited 100% of scatter activity induced by HGF (Supplementary Figure 1).

### Cell proliferation assay

(T)GCT cell lines were cultured in 12-well plates. After 8 h, they were starved for 16 h under serum-free conditions and then cultured in medium containing 2% FBS and HGF for 24, 48 and 72 h. PF-04217903 was also used alone or in combination with HGF. After indicated culture times, cells were trypsinized, harvested and counted.

### Cell cycle FACS analysis

Cells were plated and after 8 h they were maintained under serum-free conditions for 16 h, to synchronize the cellular cycle. Then, cells were cultured for 48 h in the absence or in the presence of HGF, PF-04217903 or both factors in 2% FBS. The cells were recovered, fixed overnight in 70% EtOH at 4° C and stained with propidium iodide (50 µg/mL)/RNase (100 U/mL) solution (Sigma-Aldrich, cat. P4864 and R6513 respectively) for at least 3 h. The cell suspensions were analyzed with an Epics XL Flow Cytometer (Beckman Coulter). The data were analyzed with the FCS Express 5.1 software (*De Novo*).

### Boyden chamber assay

Chemotaxic migration of cells was assayed using a polycarbonate filter (pore size, 8 µm, Whatman International, cat. 150446) positioned in Boyden chambers (Neuro-Probe, Gaithersburg, MD, USA). Cells were starved for 16 h under serum-free conditions and then 5 × 10^4^ cells/well were added to the upper chamber, whereas in the lower chamber, HGF or 1% FBS (as positive control) were added as chemo-attractants. To abolish HGF gradient, the factor was added both to the upper and the lower chambers. After 5 h, the polycarbonate filters were harvested, cells on the upper surface were mechanically removed, filters were fixed with paraformaldehyde 4% in PBS (pH 7.4) at 4° C, and stained with Diff Quick solution (DADE, cat. 130832). The results are reported as the fold increases.

### Matrigel invasion assay

TGCT cell invasion was assayed using chambers coated with Matrigel (Basement Membrane Matrix Growth Factor Reduced, BD Biosciences, cat. 354483). The cells (2,5 × 10^4^/well) were cultured on Matrigel layer in medium containing HGF, PF-04217903 or both factors in 2% FBS. After 24 h, cells on the upper surface were mechanically removed, Matrigel layer containing invading cells was fixed with paraformaldehyde (4%, w/v) and stained with Diff Quick solution. Four chamber fields were photographed, the average number ± SEM of cells per field was calculated and reported as the fold increase. MMP inhibition was evaluated only in NT2D1 cells. The cells cultured on Matrigel layer were treated with GM6001 alone or in combinations with HGF and then processed as described above.

### Gel zymography

All TGCT cell lines were cultured for 8 h, serum-starved for 16 h and then treated with HGF, PF-04217903 or both factors in 2% FBS for 24, 48 and 72 h. For uPA zymography, equal amounts of conditioned media were separated under non-reducing conditions in a 10% SDS-PAGE. The gel was washed twice in 2.5% Triton X-100 (Sigma-Aldrich, cat. X-100) and then placed on a casein-agar layer containing human plasminogen as a substrate (Sigma-Aldrich, cat. P7999). Gelatinolytic activity of conditioned media was assayed as previously described [55]. Briefly, 20 µl aliquots of conditioned media and cell extracts were fractionated by 10%SDS-PAGE in the presence of 0.1% gelatin under non-reducing conditions. Following gel electrophoresis, the gels were washed twice in 2.5% TritonX-100 for 30 min at room temperature to remove SDS. The gels were incubated at 37°C overnight in substrate buffer, stained with 0.5% Coomassie Brilliant Blue R250 and de-stained in 30% methanol and 10% glacial acetic acid (vol/vol). Precision Plus Protein All Blue Standards (Bio-Rad Laboratories) have been used as molecular weight markers. Densitometric analysis of bands was performed using the ImageJ software. Mean ± SEM are reported.

### Patient samples

Multiple Tissue Micro-Arrays (TMA) were investigated, each including primary TGCTs of pure and mixed histology. The samples were obtained prior to possible systemic therapy. Representative areas, identified by both morphological characterization as well as additional immunohistochemical staining (including OCT3/4, SOX17, SOX2, CD30, AFP and hCG), were selected by an experienced pathologist (JWO). These specifically identified various histological elements, including GCNIS, seminoma, and the various types of non-seminomas, i.e., embryonal carcinoma, yolk sac tumor and choriocarcinoma. Use of tissue samples remaining after diagnosis for scientific reasons was approved by the Medical Ethical Committee (MEC) of the Erasmus MC Rotterdam (The Netherlands), permission 02.981. This included the permission to use the secondary tissue without further consent. Samples were used according to the “Code for Proper Secondary Use of Human Tissue in The Netherlands” developed by the Dutch Federation of Medical Scientific Societies (FMWV (Version 2002, update 2011)).

### C-MET immunohistochemical staining of TMA sections and whole tissue sections

Tissue slides were deparaffinized and rehydrated. Endogenous peroxidase activity was inactivated in 3% H_2_O_2_ for 5 min. Subsequently, the slides were subjected to antigen retrieval procedure by heating them under high pressure up to 1.2 bar in Tris-EGTA buffer (0.01 M Tris, 0.001 M EGTA, pH 9.0). Endogenous biotin was blocked using the Avidin/Biotin Blocking kit (Vector Laboratories Ltd; cod. SP-2001). Slides were incubated overnight at 4° C with the primary rabbit c-MET (D1C2) antibody (Cell Signaling, 1:100, cat. 8198). After washing in PBS-Tween 1%, the slides were incubated for 30 min at room temperature with the secondary biotinylated polyclonal anti-rabbit antibody (1:150; Dako cod. E0431), and the signal was amplified using the VECTASTAIN Elite ABC system (Vector Laboratories Ltd, cod. PK-6100). Finally, horseradish peroxidase activity was visualized with 3, 3′-diaminobenzidine (Dako, cod. K3468) prepared following the manufacturer›s instructions. Nuclei were stained with hematoxylin solution. Staining intensities were evaluated separately for nuclear, cytoplasmic and membranous immunoreactivity on a scale from - to +++. Two observers (JWO and KCS) assessed the immunoreactivity scores.

### Statistical analysis

All quantitative data are presented as the mean ± standard error (SEM). Student’s *t*-test and an ANOVA for multi-group comparison were carried out. All experiments were performed at least in triplicate, and the significance level was fixed at a *p* value < 0.05.

## SUPPLEMENTARY MATERIALS FIGURES



## Abbreviations

BCA: Bicinchoninic Acid assay
BSA: Bovine Serume Albumine
CCNB1: Cyclin B1
CHC: Choriocarcinoma
c-MET: Mesenchymal Epithelial Transition factor (HGF receptor)
EC: Embryonal Carcinoma
FBS: Foetal Bovine Serum
FISH: Fluorescence *In Situ* Hybridization
GAPDH: Glyceraldehyde-3-phosphate dehydrogenase
GCNIS: Germ Cell Neoplasia *In Situ*
HGF: Hepatocyte Growth Factor
HPRT1: Hypoxanthine phosphoribosyltransferase 1
MDCK: Madin-Darby Canine Kidney
MEC: Medical Ethical Committee
MMPs: metalloproteinases
SE: Seminoma
SEM: Standard Error Measure
TE: Teratoma
TGCTs: Testicular Germ Cell Tumours
TMA: Tissue Microarray
uPA: urokinase Plasminogen Activator
YST: Yolk Sac Tumour
