# Correction: c-MET receptor as potential biomarker and target molecule for malignant testicular germ cell tumors

**DOI:** 10.18632/oncotarget.26374

**Published:** 2018-11-13

**Authors:** Katia Corano Scheri, Erica Leonetti, Luigi Laino, Vincenzo Gigantino, Luisa Gesualdi, Paola Grammatico, Mariano Bizzarri, Renato Franco, J. Wolter Oosterhuis, Hans Stoop, Leendert H.J. Looijenga, Giulia Ricci, Angela Catizone

**Affiliations:** ^1^ Department of Anatomy, Histology, Forensic-Medicine and Orthopaedics, “Sapienza” University of Rome, Italy; ^2^ Department of Molecular Medicine, Laboratory of Medical Genetics, “Sapienza” University of Rome, San Camillo-Forlanini Hospital, Rome, Italy; ^3^ Pathology Unit, Istituto Nazionale Tumori I.R.C.C.S. “Fondazione Pascale”, Naples, Italy; ^4^ Department of Experimental Medicine, Systems Biology Group Lab, “Sapienza” University of Rome, Italy; ^5^ Pathological Anatomy Unit, Department of Psychic and Physic health and preventive medicine, Università degli Studi della Campania “Luigi Vanvitelli”, Naples, Italy; ^6^ Department of Pathology, Laboratory for Experimental Patho-Oncology, Erasmus MC University Medical Center, Cancer Institute, Rotterdam, The Netherlands; ^7^ Department of Experimental Medicine, Università degli Studi della Campania “Luigi Vanvitelli”, Naples, Italy

**This article has been corrected:** The correct author name is given below:

**Mariano Bizzarri**

Original article: Oncotarget. 2018; 9:31842-31860. https://doi.org/10.18632/oncotarget.25867

