# Supplementary material for: c-MET receptor as potential biomarker and target molecule for malignant testicular germ cell tumors
Source: Oncotarget. 2018 Aug 7;9(61):31842–60. doi: 10.18632/oncotarget.25867 (PMC6112764; doi:10.18632/oncotarget.25867)
Supplement: Supplementary file 1 [file oncotarget-09-31842-s001.pdf]

c-MET receptor as potential biomarker and target molecule for malignant testicular germ cell tumor

SUPPLEMENTARY MATERIALS

A

HGF and PF-04217903 doses	MDCK Scattering phenotype
CTRL .	-
HGF 10 ng/ml	+/-
HGF 20 ng/ml	++
HGF 40 ng/ml	+++
HGF 60 ng/ml	+++
HGF 40 ng/ml + 5nM PF-04217903	+++
HGF 40 ng/ml + 10nM PF-04217903	+++
HGF 40 ng/ml + 20nM PF-04217903	++
HGF 40 ng/ml + 30nM PF-04217903	+/-
HGF 40 ng/ml + 10µM PF-04217903	-
HGF 40 ng/ml + 50µM PF-04217903	-

B

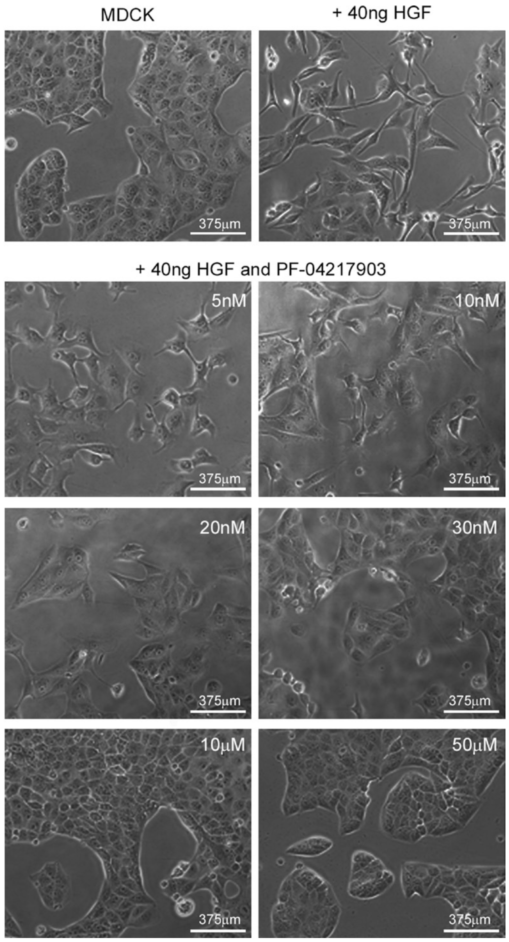

Supplementary Figure 1: Dose-response of HGF scatter activity on MDCK colonies. (**A**) Summary table of scoring analysis of dose-response of HGF and HGF+PF-04217903 scatter activity on MDCK cells. (**B**) In the upper part of the panel: representative images of cluster morphology of MDCK cells, un-stimulated or stimulated with 40 ng/ml of HGF. In the lower part: representative images of the abrogating effect of PF-04217903 on scattering phenotype induced by HGF (scale bar: 375 µm).

A

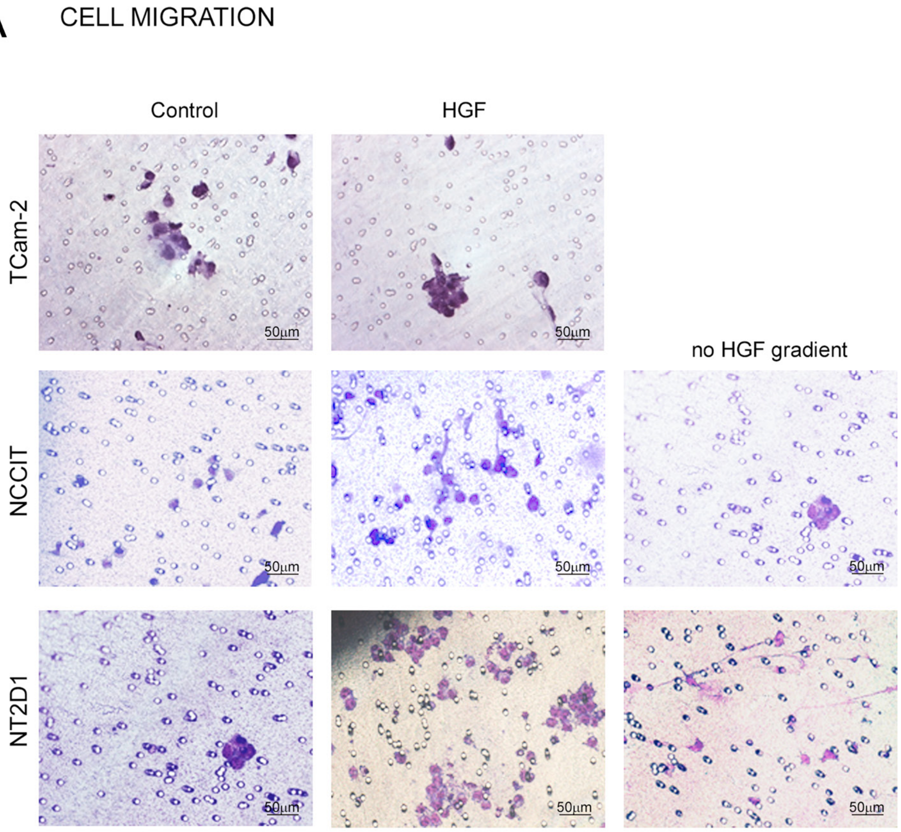

B

Cell line	Treatments	Cells/filter (Mean)	SEM	St Dev
TCam-2	Control	61	12	29
	1% FBS	174	20	48
	HGF	92	3	7
NCCIT	Control	141	14	41
	1% FBS	342	58	129
	HGF	353	21	63
	no HGF gradient	153	12	24
NT2D1	Control	176	6	26
	1% FBS	389	15	61
	HGF	346	15	58
	no HGF gradient	197	6	26

Supplementary Figure 2: Short term migration in (T)GCT cell lines upon HGF stimulation. (**A**) Representative images of migrated cells on policarbonate filter in control condition (serum free medium + 0.1%BSA) and after 5 h of HGF chemo-attraction of TCam-2, NCCIT and NT2D1 cells. Representative images of migrated cells when HGF gradient is abrogated are also reported for NCCIT and NT2D1 samples (scale bar: 50 μm). (**B**) Representative table with the number of migrated cells/filter for each cell line in control condition and after 5 h of HGF chemo-attraction. Mean ± SEM and SD are reported.

A

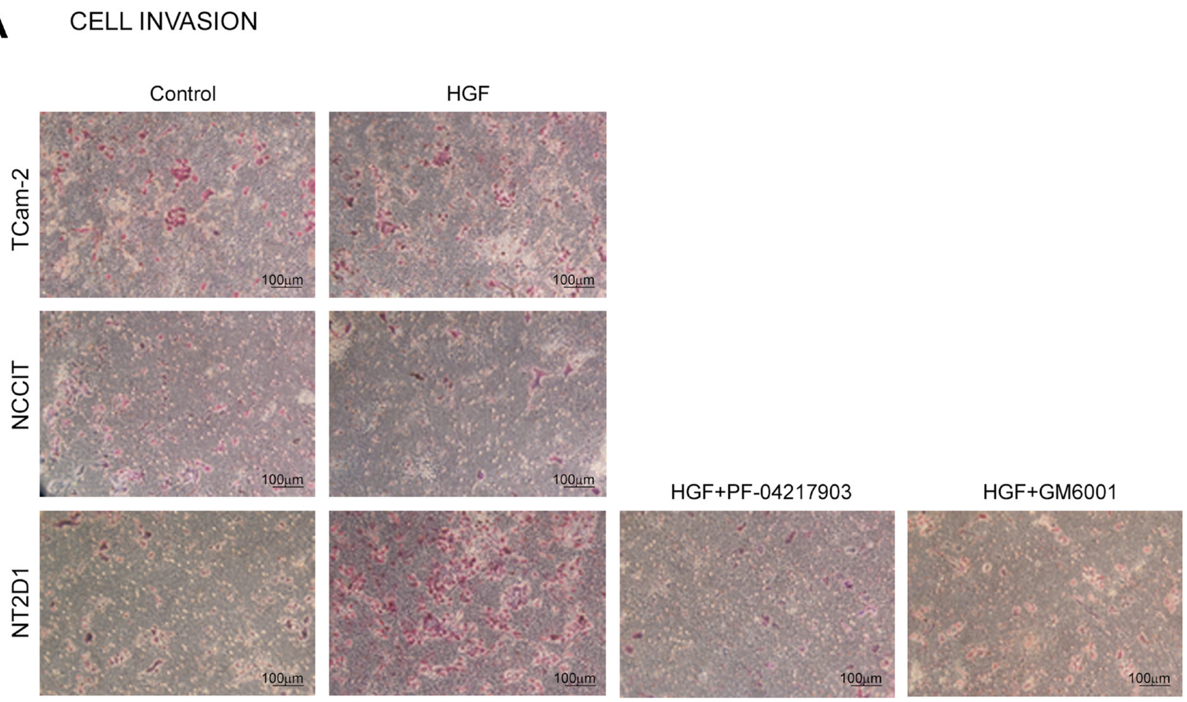

B

Cell line	Treatments	Cells/field (Mean)	SEM	St Dev
TCam-2	Control	58	4	12
	HGF	62	5	14
NCCIT	Control	66	6	11
	HGF	85	8	14
NT2D1	Control	40	4	18
	HGF	75	3	19
	PF-0427903	37	2	6
	HGF+PF04217903	39	2	10
	GM6001	55	9	16

Supplementary Figure 3: Matrigel invasion assay in (T)GCT cell lines upon HGF stimulation. (**A**) Representative images of invading cells in control condition (medium + 2% FBS + 0.1%BSA) and after 24 h of HGF stimulation of TCam-2, NCCIT and NT2D1 cell lines. For NT2D1 cells representative images of HGF+PF-04217903 and HGF+GM-6001 treatments are also reported (scale bar: 100 µm). (**B**) Representative table with the number of invading cells/field for each cell line in control condition and after HGF, HGF+PF-04217903 and HGF+GM-6001 stimulation. Mean ± SEM and SD are reported.

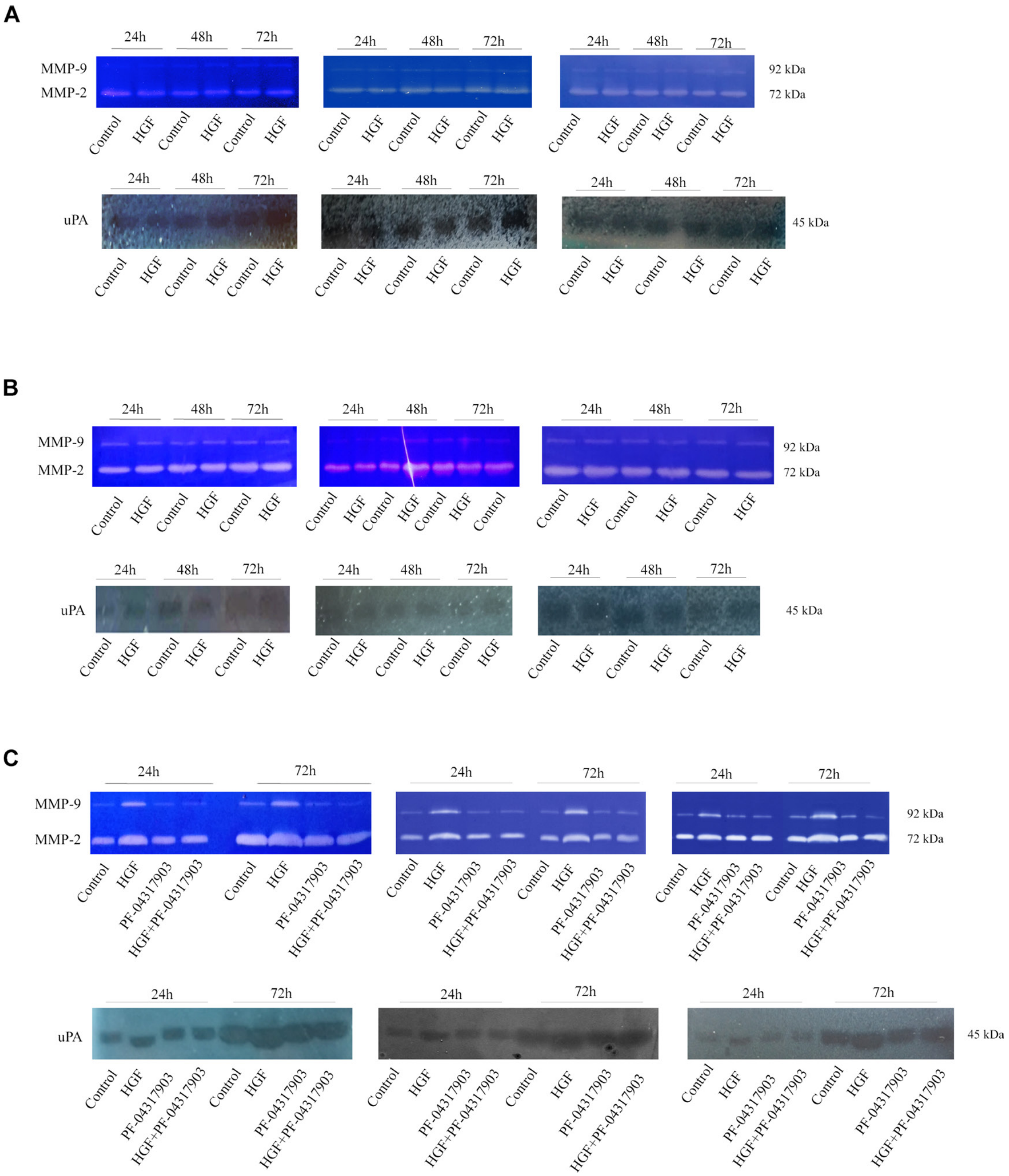

Supplementary Figure 4: uPA and MMP activities in GCT conditioned media. Images of the gel zymographies for uPA and MMP (MMP-2/MMP-9) enzymatic activity in TCam-2 (**A**), NCCIT (**B**) and NT2D1 (**C**) conditioned media in control condition and after 24, 48 and 72 h from HGF administration. For NT2D1 cells representative images of uPA and MMP activity in PF-04217903 and HGF+PF-04217903 administered culture media is also reported.

c-MET relative expression (a.u.)	Mean	SEM	St Dev
TCam-2	1,60	0,16	0,32
NCCIT	1,00	0,03	0,06
NT2D1	1,25	0,02	0,05
MCF10	2,12	0,17	0,35

c-MET expression (WB) (a.u.)	Mean	SEM	St Dev
TCam-2	0,42	0,07	0,15
NCCIT	0,62	0,07	0,13
NT2D1	0,67	0,08	0,15

Proliferation assay (Cell number)	Mean	SEM	St Dev
**24h TCam-2**			
Control	9,05	0,37	0,64
HGF	9,32	0,31	0,54
**24h NCCIT**			
Control	10,34	0,36	0,63
HGF	11,03	0,36	0,63
**24h NT2D1**			
Control	9,91	0,40	0,71
HGF	10,33	0,13	0,23
PF-04217903	9,53	0,13	0,23
HGF+PF-04217903	9,86	0,12	0,21
**48h TCam-2**			
Control	11,74	0,48	0,84
HGF	12,55	0,27	0,48
**48h NCCIT**			
Control	13,55	0,49	0,85
HGF	14,40	0,15	0,27
**48h NT2D1**			
Control	13,54	0,13	0,34
HGF	15,50	0,15	0,32
PF-04217903	13,46	0,03	0,06
HGF+PF-04217903	13,67	0,36	0,63
**72h TCam-2**			
Control	12,86	0,44	0,78
HGF	14,22	0,22	0,38
**72h NCCIT**			
Control	17,22	0,48	0,84
HGF	19,36	0,81	1,41
**72h NT2D1**			
Control	17,33	0,68	1,13
HGF	18,76	0,48	0,84
PF-04217903	16,76	0,49	0,85
HGF+PF-04217903	17,43	0,34	0,64

NT2D1 Cell cycle analysis (% G2/M)	Mean	SEM	St Dev
Control	16,66	0,32	1,11
HGF	22,17	0,81	2,80
PF-04217903	19,34	0,71	2,44
HGF+PF-04217903	16,63	0,56	1,95

NT2D1 CCNB1 relative expression	Mean	SEM	St Dev
T0	1,00	0,04	0,06
3h			
Control	0,47	0,14	0,24
HGF	0,86	0,11	0,19
6h			
Control	0,87	0,08	0,21
HGF	0,71	0,11	0,28
12h			
Control	0,90	0,14	0,31
HGF	0,99	0,11	0,28
18h			
Control	1,27	0,19	0,38
HGF	0,96	0,18	0,37
24h			
Control	1,00	0,19	0,67
HGF	2,37	0,17	0,30
PF-04217903	1,24	0,23	0,40
HGF+PF-04217903	1,30	0,34	0,59
48h			
Control	0,70	0,06	0,16
HGF	0,83	0,10	0,26
54h			
Control	0,40	0,06	0,12
HGF	1,14	0,03	0,05

NT2D1 c-MET relative expression	Mean	SEM	St Dev
T0	1,00	0,08	0,11
3h			
Control	2,59	0,98	1,69
HGF	6,05	1,82	3,15
6h			
Control	14,81	1,36	8,09
HGF	9,77	2,20	2,53
12h			
Control	3,44	1,34	2,33
HGF	21,44	2,35	4,07
18h			
Control	23,52	3,18	5,50
HGF	46,26	4,40	18,01
24h			
Control	16,73	4,54	7,87
HGF	21,17	4,85	8,39
48h			
Control	11,00	1,93	3,34
HGF	14,66	3,34	4,73

NT2D1 c-MET expression (WB)	Mean	SEM	St Dev
T0	0,78	0,10	0,20
8h			
Control	1,39	0,12	0,20
HGF	0,66	0,06	0,10
12h			
Control	1,04	0,08	0,13
HGF	0,73	0,06	0,10
18h			
Control	1,42	0,17	0,30
HGF	0,28	0,00	0,01
24h			
Control	1,05	0,18	0,37
HGF	0,38	0,10	0,20
PF-04217903	1,11	0,10	0,22
HGF+PF-04217903	1,34	0,15	0,29
48h			
Control	1,30	0,09	0,16
HGF	0,25	0,07	0,13
PF-04217903	0,80	0,10	0,21
HGF+PF-04217903	0,60	0,10	0,20

Supplementary Figure 5: Statistical values relative to Figures 1, 3 and 7. Tables illustrating all the mean values, presented in the graphs of Figures 1, 3 and 7, together with their SEM and SD.

Boyden assay (cell number/filter)	Mean	SEM	St Dev
**TCam-2**			
Control	1,00	0,19	0,36
1% FBS	2,84	0,32	0,79
HGF	1,50	0,05	0,11
**NCCIT**			
Control	1,00	0,09	0,19
1% FBS	2,43	0,33	0,57
HGF	2,51	0,14	0,33
no HGF gradient	1,08	0,09	0,17
**NT2D1**			
Control	1,00	0,04	0,14
1% FBS	2,22	0,14	0,35
HGF	1,97	0,11	0,29
no HGF gradient	1,12	0,07	0,15

uPA activity (24h)	Mean	SEM	St Dev
**TCam-2**			
Control	1,00	0,03	0,07
HGF	1,02	0,01	0,03
**NCCIT**			
Control	1,00	0,05	0,17
HGF	1,22	0,01	0,02
**NT2D1**			
Control	0,99	0,01	0,02
HGF	1,59	0,09	0,15
PF-04217903	1,06	0,07	0,12
HGF+PF-04217903	0,96	0,03	0,06

MMP-9 activity (24h)	Mean	SEM	St Dev
**TCam-2**			
Control	2129,67	321,07	556,12
HGF	2210,67	270,28	468,14
**NCCIT**			
Control	7,35	0,56	1,15
HGF	7,10	1,50	3,10
**NT2D1**			
Control	5318,50	627,60	1087,03
HGF	33090,60	1582,47	2740,92
PF-04217903	5516,66	313,27	542,61
HGF+PF-04217903	5998,33	570,97	988,96

MMP-9 activity (48h)	Mean	SEM	St Dev
**TCam-2**			
Control	2009,67	156,10	270,37
HGF	2148,33	156,02	270,23
**NCCIT**			
Control	9,58	1,59	3,60
HGF	7,75	0,95	1,98

MMP-2 activity (72h)	Mean	SEM	St Dev
**TCam-2**			
Control	3967,17	483,9	838,06
HGF	4215,75	570,7	988,49
**NCCIT**			
Control	17,28	0,563	1,20
HGF	18,47	0,161	0,26
**NT2D1**			
Control	21087,33	934	12294,53
HGF	33825,33	2632	18511,48
PF-04217903	22938,33	2027	14286,33
HGF+PF-04217903	21815,67	1457	13255,75

Invasion assay (cell number/field)	Mean	SEM	St Dev
**TCam-2**			
Control	1,00	0,06	0,21
HGF	1,09	0,12	0,66
**NCCIT**			
Control	1,00	0,09	0,19
HGF	1,30	0,13	0,64
**NT2D1**			
Control	1,00	0,11	0,46
HGF	1,62	0,12	0,46
PF-04217903	0,91	0,04	0,16
HGF+PF-04217903	0,96	0,06	0,24
GM6001	1,39	0,22	0,39
HGF+GM6001	1,50	0,08	0,14

MMP-2 activity (24h)	Mean	SEM	St Dev
**TCam-2**			
Control	3007,08	222	384,95
HGF	3247,08	287	496,84
**NCCIT**			
Control	16,44	1	2,60
HGF	14,31	1	2,80
**NT2D1**			
Control	17389,33	438	759,47
HGF	40272,00	581	1006,93
PF-04217903	19856,00	1014	1756,74
HGF+PF-04217903	22483,00	381	660,23

uPA activity (48h)	Mean	SEM	St Dev
**TCam-2**			
Control	1,00	0,06	0,13
HGF	1,02	0,03	0,05
**NCCIT**			
Control	1,00	0,06	0,13
HGF	1,13	0,03	0,06

MMP-2 activity (48h)	Mean	SEM	St Dev
**TCam-2**			
Control	3326,41	212,3	367,64
HGF	3472,16	287,7	498,26
**NCCIT**			
Control	17,26	1,13	2,5
HGF	16,23	1,46	2,78

uPA activity (72h)	Mean	SEM	St Dev
**TCam-2**			
Control	1,00	0,03	0,07
HGF	1,07	0,01	0,02
**NCCIT**			
Control	1,00	0,03	0,07
HGF	1,12	0,01	0,02
**NT2D1**			
Control	1,04	0,03	0,05
HGF	1,56	0,01	0,02
PF-04217903	1,04	0,04	0,06
HGF+PF-04217903	1,01	0,02	0,03

MMP-9 activity (72h)	Mean	SEM	St Dev
**TCam-2**			
Control	2049,67	62,23	107,78
HGF	2200,00	120,40	208,54
**NCCIT**			
Control	9,50	1,51	3,25
HGF	8,70	0,20	0,50
**NT2D1**			
Control	6875,50	982,06	1700,98
HGF	29473,00	2212,81	3832,69
PF-04217903	7560,00	1084,72	1878,79
HGF+PF-04217903	6090,60	1485,68	2573,27

Supplementary Figure 6: Statistical values relative to Figures 4, 5 and 6. Tables illustrating all the mean values, presented in the graphs of Figures 4, 5 and 6, together with their SEM and SD.
